# Pharmacological characterization of DA‐7503: a novel tau aggregation inhibitor in clinical development for Alzheimer’s disease and primary tauopathies

**DOI:** 10.1002/alz70859_100012

**Published:** 2025-12-25

**Authors:** Min Jung Lee, Sungsu Lim, Yun Kyung Kim, Kea Joo Lee, Bo‐Ram Lee, Soo‐Jung Choi, Hyangsoo Lee, Jun‐Hwan Moon, Mi‐Kyung Kim, Ae Nim Pae

**Affiliations:** ^1^ Dong‐A ST Research Headquarter, Yongin Korea, Republic of (South); ^2^ Korea Institute of Science and Technology, Seoul Korea, Republic of (South); ^3^ Korea Institute of Science and Technology, Seoul, Seoul Korea, Republic of (South); ^4^ Korea Brain Research Institute, Daegu Korea, Republic of (South)

## Abstract

**Background:**

DA‐7503 is a potential first‐in‐class, orally available small‐molecule inhibitor of tau oligomerization, currently in clinical development for the treatment of Alzheimer’s disease and primary tauopathies. The neurobehavioral and biochemical findings from AD and tauopathy mouse models revealed that DA‐7503 improved memory and recognition, and reduced tau aggregation and hyperphosphorylation in the brain. This study aimed to elucidate the pharmacological properties of DA‐7503, focusing on its potency and specificity for pathological tau isoforms, as well as its therapeutic effects on motor impairments and tau aggregation in the JNPL3 mouse model.

**Method:**

The inhibitory potency of DA‐7503 on aggregation of 4R and 3R tau isoforms was evaluated in tau‐BiFC HEK293 cells. For in vivo studies, JNPL3 transgenic mice, expressing human P301L‐mutant tau, were administrated with DA‐7503 orally once daily for 4.5 months from 8 months of age. Therapeutic effects were assessed through motor impairment evaluations and analysis of pathological tau aggregation. Non‐clinical toxicity and safety pharmacology studies, including 28‐day rat and dog toxicity assessments, were conducted according to GLP principles.

**Result:**

This study reports the non‐clinical characteristics of DA‐7503, a novel tau aggregation inhibitor, in terms of efficacy and safety. DA‐7503 demonstrated comparable inhibitory potency against aggregation of both 4R tau and 3R tau under various tau aggregation‐inducing conditions in tau‐BiFC cells. In JNPL3 mice, DA‐7503 treatment significantly improved motor function in several behavioral tests, including the open field, coat hanger, vertical grid, rotarod, and gait pattern analysis. Notably, a dose of 15 mg/kg was sufficient to reduce pathological tau aggregates in the brainstem and spinal cord of JNPL3 mice. Non‐clinical toxicity studies revealed that DA‐7503 has a favorable safety profile, supporting its clinical development.

**Conclusion:**

Our findings suggest that DA‐7503, a novel tau aggregation inhibitor with great potencies against pathological 3R and 4R tau isoforms and a favorable non‐clinical safety profile, effectively restored motor function and reduced tau aggregates in the JNPL3 mouse model of tauopathy. These results highlight DA‐7503’s potential as a promising therapeutic candidate for Alzheimer’s disease and primary tauopathies, including frontotemporal dementia, progressive supranuclear palsy and corticobasal degeneration. DA‐7503 is currently undergoing a phase 1 clinical study.

